# Prosthodontic Rehabilitation of the Patient with Severely Worn Dentition: A Case Report

**DOI:** 10.1155/2012/961826

**Published:** 2012-07-09

**Authors:** Mahnaz Hatami, Mahmoud Sabouhi, Siamak Samanipoor, Hamid Badrian

**Affiliations:** ^1^Department of Prosthodontics, School of Dentistry, Shahid Sadooghi University of Medical Sciences, Yazd 8914881167, Iran; ^2^Department of Prosthodontics, School of Dentistry, Isfahan University of Medical Sciences, Isfahan, Iran; ^3^Department of Endodontics, School of Dentistry, Shahid Bahonar University of Medical Sciences, Kerman, Iran; ^4^Dental Implant Research Center, School of Dentistry, Isfahan University of Medical Sciences, Isfahan, Iran

## Abstract

The management of tooth wear has been a subject of increasing interest from both preventive and restorative points of view. This paper describes the full mouth rehabilitation of a 63-year-old bruxer man with a severely worn dentition and other dental problems including unsuitable restorations and several missing teeth. The treatment entailed using cast posts and cores, metal-ceramic restorations, and a removable partial denture. As with the treatment procedure of such cases, equal-intensity centric occlusal contacts on all teeth and an anterior guidance in harmony with functional jaw movements were especially taken into account.

## 1. Introduction

Severe tooth wear is a potential threat for dentition and masticatory function. Many factors may combine to produce the worn dentition, and the etiology often remains unidentified [1]. Tooth wear has been classified into the following four types: (1) attrition, which is the wear of teeth or restorations caused by tooth to tooth contact during mastication or parafunction; (2) abrasion, which is the loss of tooth surface caused by abrasion with foreign substances other than tooth to tooth contact; (3) erosion, which is the loss of tooth surface by chemical processes not involving bacterial action; (4) abfraction, that is noncarious cervical wedge-shaped defect caused by occlusal stresses [2–4].

The management of tooth wear, especially attrition, is becoming a subject of increasing interest in the prosthodontics literature, from both preventive and restorative points of view [5]. A critical aspect for successful treatment is to determine the occlusal vertical dimension (OVD) and the interocclusal rest space (IRS). A systematic approach for managing tooth wear can lead to a predictable and favourable prognosis [6].

This paper presents the stages of prosthodontic rehabilitation, from diagnosis to final treatment and followup, of a bruxer patient with severely worn dentition, some extracted teeth and uneven occlusal plane, using casted posts and cores, metal-ceramic restorations, removable partial denture (RPD), and an occlusal splint for protecting the restorations from patient's parafunction.

## 2. Case Report

### 2.1. Examination

**Figure 1:**
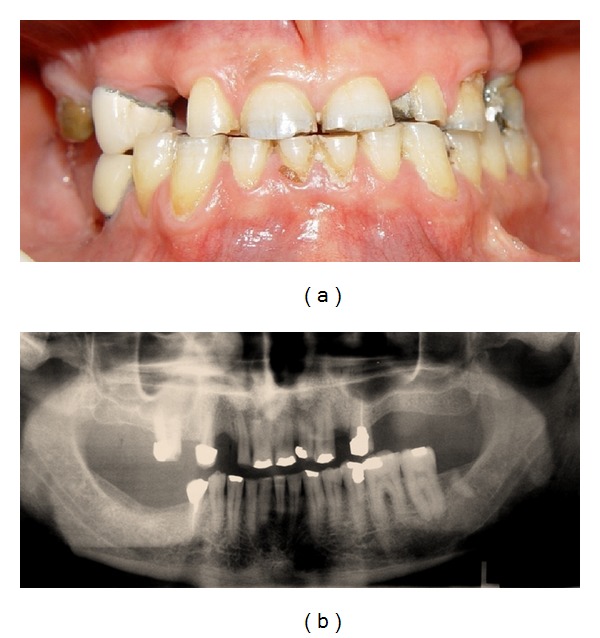
Frontal view of teeth in occlusion (a) and panoramic radiograph (b) before treatment.

A 63-year-old man was referred to the Department of Prosthodontics in the Faculty of Dentistry at Isfahan University of Medical Sciences, Iran, for prosthodontic treatment. The patient's chief complaint was the restoration of worn teeth, in addition to the replacement of unacceptable restorations and missing teeth. An initial evaluation of the patient indicated a history of depression, and also, parafunctional habits of bruxism and clenching. Oral hygiene was fair, and there was no periodontal problem. Clinical and radiographic examinations and diagnostic casts revealed severe attrition, especially on anterior teeth and an uneven occlusal plane (Figure 1). The causes of the severe wear were parafunctional habits, unsuitable restorations, and a lack of stable posterior occlusion.

### 2.2. Treatment

**Figure 2:**
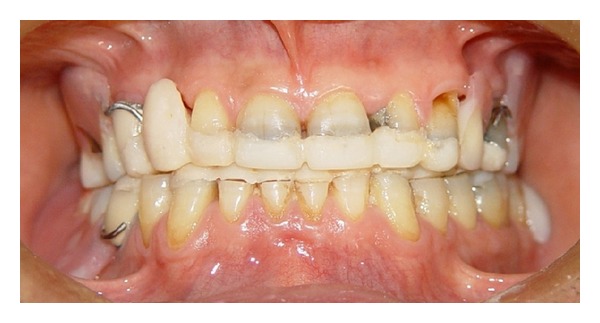
Removable provisional prostheses in place.

After oral hygiene instructions, making impressions and diagnostic workup, removable provisional prostheses were fabricated with correct occlusal plane and adjusted clinically for achieving good aesthetics, phonetics, and OVD. This removable prosthesis was used to evaluate the OVD and patient tolerance (Figure 2).

**Figure 3:**
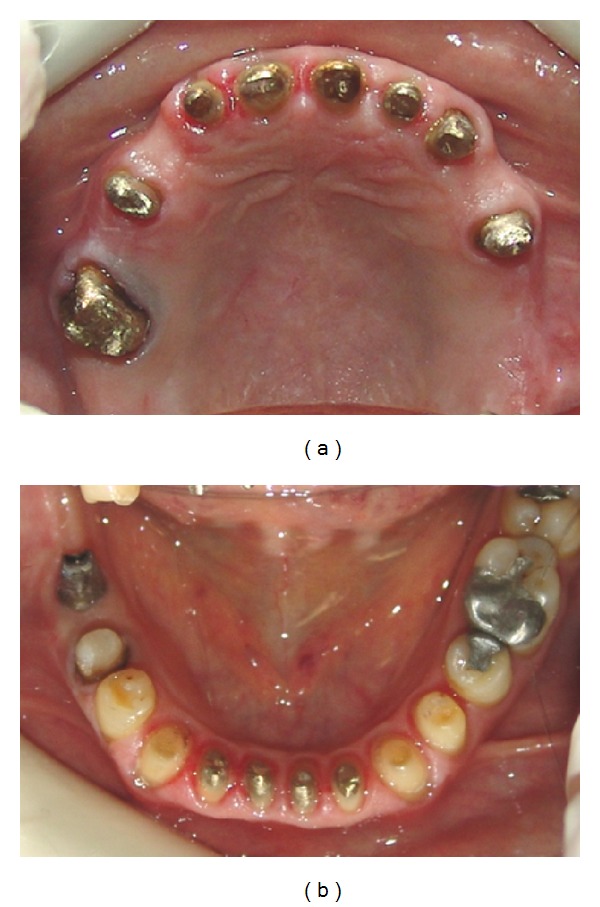
Cast posts and cores.

**Figure 4:**
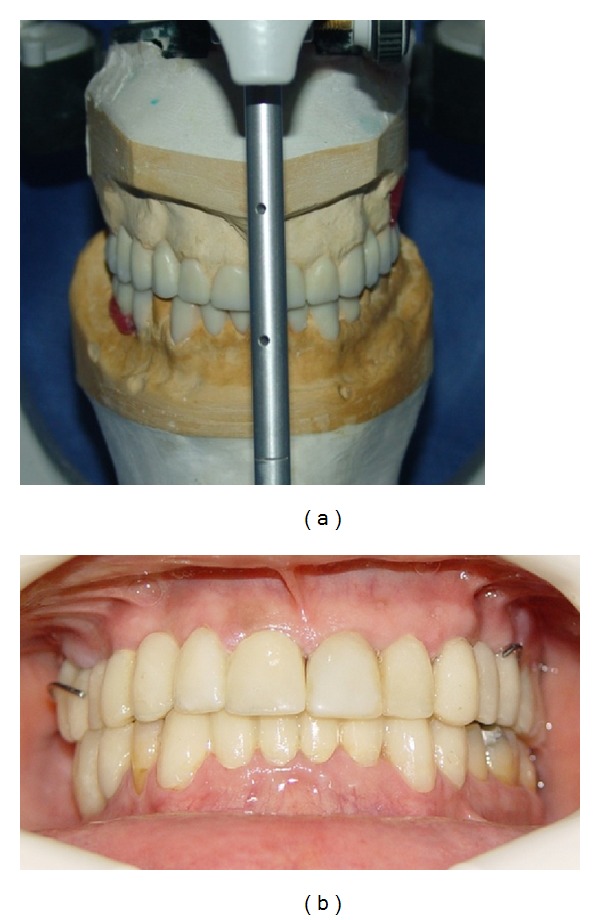
Diagnostic waxup (a); fixed and removable provisional restorations in place (b).

**Figure 5:**
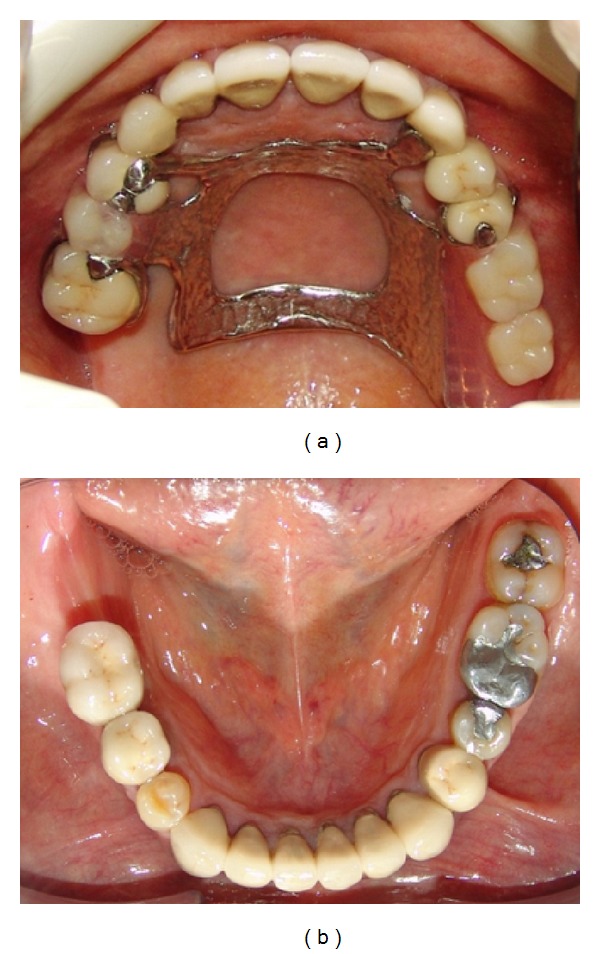
Occlusal view of the maxillary (a) and mandibular (b) arch after treatment.

**Figure 6:**
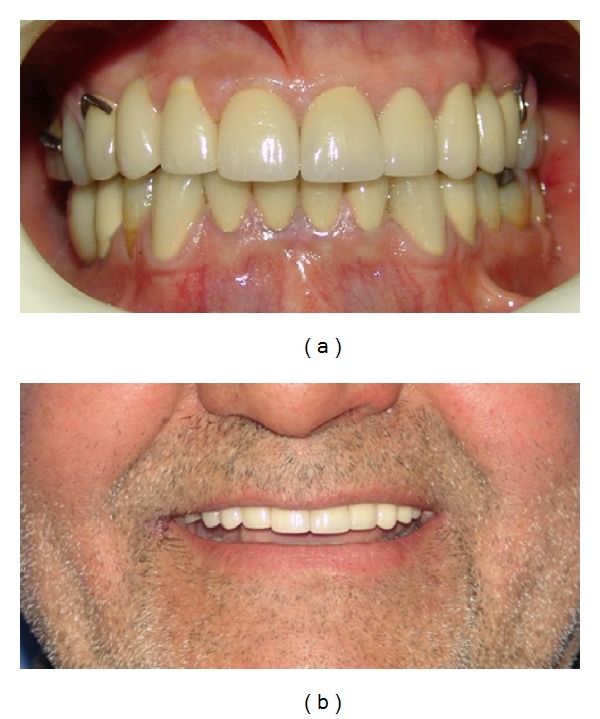
Frontal view of teeth in occlusion after treatment (a); smile view after treatment (b).

**Figure 7:**
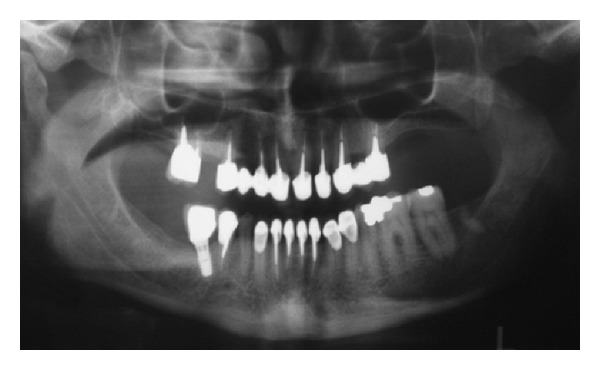
Panoramic view of 1-year-followup.

An ITI implant (4.8 × 10) (Straumann, Basel, Switzerland) was inserted in right first lower molar region after precise clinical and radiographic evaluation and diagnostic waxup using surgical stent. Root canal therapy (RCT) of anterior worn teeth and retreatment of teeth with unacceptable RCTs were performed, and casted posts and cores were fabricated (Figure 3). Fixed and removable provisional restorations were inserted and adjusted until patient acceptance achieved. These restorations were fabricated according to the diagnostic waxup (Figure 4), for which the Broadrick flag analyzer was used to determine the curve of occlusal plane. Impressions were made from provisional restorations, and casts were transferred to the Denar Mark II articulator (Teledyne Water pik, Fort Collins, CO, USA) using the Denar Slidematic facebow (Teledyne Water pik, Fort Collins, CO, USA). Then, an anterior guide table was customized by pattern resin (Duralay, Reliance Dental MFG Co., Worth, CO, USA). After completion of teeth preparations, the final impressions were made with silicon impression material (Speedex, Coltene AG, Alstatten, Switzerland/impergum, 3 M ESPE), and metal-ceramic restorations were fabricated. In the maxillary restorations, rest seats, guide planes, and retentive undercuts were formed in the RPD abutments. A Kennedy class II mode 1 maxillary RPD was fabricated and delivered (Figure 5). Finally, the occlusion of restorations was adjusted so that equal-intensity centric contacts were established on all teeth (Figure 6(a)), and anterior guidance discluded all posterior teeth in eccentric jaw movements. A maxillary occlusal splint was fabricated for protecting the restorations from patient's parafunction. The smile view of patient after treatment is shown in Figure 6. One-year followup showed no problem in teeth, restorations and temporomandibular joints and Figure 7 show the panoramic image after this period.

## 3. Discussion

In the treatment of severely worn teeth, equal-intensity centric occlusal contacts on all teeth should be accomplished. An anterior guidance should also be established in harmony with normal functional jaw movements and all posterior teeth discluded immediately during any eccentric jaw movement. If there is habitual bruxism, an occlusal splint should be delivered to the patient [7].
